# Nutrient-Based Approaches for Melanoma: Prevention and Therapeutic Insights

**DOI:** 10.3390/nu15204483

**Published:** 2023-10-23

**Authors:** Yucheng Dong, Jiaxin Wei, Fan Yang, Yang Qu, Jiuzuo Huang, Di Shi

**Affiliations:** 1 Peking Union Medical College Hospital, Chinese Academy of Medical Sciences and Peking Union Medical College, Beijing 100730, China; dongyc17@gmail.com; 2 Department of Emergency Department, Peking Union Medical College Hospital, Chinese Academy of Medical Sciences and Peking Union Medical College, Beijing 100730, China; weijiaxin729@163.com; 3 Department of Liver Surgery, Peking Union Medical College Hospital, Chinese Academy of Medical Sciences and Peking Union Medical College, Beijing 100730, China; pumc_yangfan@student.pumc.edu.cn; 4 Department of Breast Surgery, Peking Union Medical College Hospital, Chinese Academy of Medical Sciences and Peking Union Medical College, Beijing 100730, China; quyang1016@163.com

**Keywords:** melanoma, diet, nutrients, prevention, therapy

## Abstract

Melanoma, a prevalent and lethal form of skin cancer, remains a formidable challenge in terms of prevention and treatment. While significant progress has been made in understanding its pathogenesis and treatment, the quest for effective prevention strategies and therapeutic approaches remains ongoing. Considering the increased advancements in understanding the dynamic interplay between nutrients and melanoma, we aim to offer a refreshed perspective on nutrient-based approaches for melanoma prevention and adjunctive therapy. In contrast to other studies, we have innovatively provided a detailed exposition of the nutrients’ influences on melanoma prognosis and treatment. This review firstly examines various nutrients, including antioxidants (namely vitamins A, D, C, and E; selenium; and caffeine), polyunsaturated fatty acids, and flavonoids, for their effects and underlying mechanisms in reducing melanoma risk. Among these nutrients, caffeine shows the most promising potential, as it is supported by multiple cohort studies for its protective effect against melanoma. In contrast, there is a certain degree of inconsistency in the research of other nutrients, possibly due to inherent differences between animal studies and epidemiological research, as well as variations in the definition of nutrient intake. To comprehensively investigate the impact of nutrients on melanoma progression and therapeutic approaches, the following sections will explore how nutrients influence immune responses and other physiological processes. While there is robust support from cell and animal studies regarding the immunomodulatory attributes of vitamins D and zinc, the anti-angiogenic potential of polyphenols, and the cell growth-inhibitory effects of flavonoids, the limited availability of human-based research substantially constrains their practical relevance in clinical contexts. As for utilizing nutrients in adjuvant melanoma treatments, multiple approaches have garnered clinical research support, including the utilization of vitamin D to decrease the postoperative recurrence rates among melanoma patients and the adoption of a high-fiber diet to enhance the effectiveness of immunotherapy. In general, the effects of most nutrients on reducing the risk of melanoma are not entirely clear. However, several nutrients, including vitamin D and dietary fiber, have demonstrated their potential to improve the melanoma prognosis and enhance the treatment outcomes, making them particularly deserving of clinical attention. A personalized and interdisciplinary approach, involving dermatologists, oncologists, nutritionists, and researchers, holds the promise of optimizing melanoma treatment strategies.

## 1. Introduction

Melanoma, an aggressive form of skin cancer originating from melanocytes, represents a critical challenge in dermatology and oncology. Its propensity for early metastasis and association with a high cancer death rate of about 55,500 per year [1] underscore the need for novel approaches for its treatment. The melanoma incidence rate is rising in developed, predominantly fair-skinned countries, growing over 320% in the US since 1975. On the other hand, its mortality rate in the US has fallen by almost 30% over the past decade with the approval of 10 new targeted or immunotherapy agents, combined with other treatment approaches. The age-standardized incidence rate is 3.8/100,000 for males and 3.0/100,000 for females, with cumulative lifetime risk rates of 0.42% and 0.33%, respectively [2].

**Figure 1:**
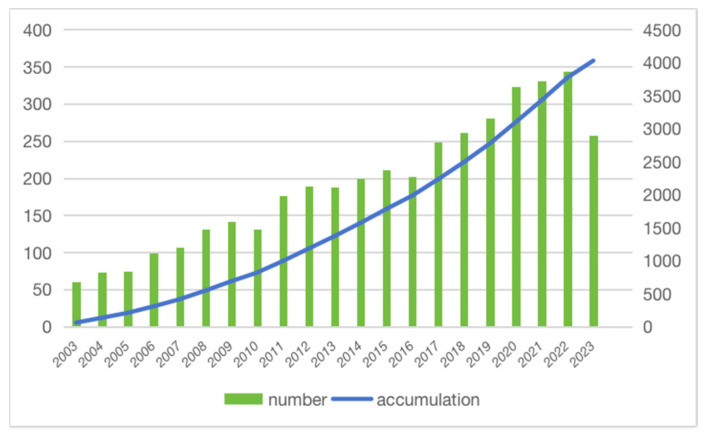
The number of publications per year and the cumulative number. The number of articles related to melanoma and nutrition showed an increasing trend year by year and the cumulative number increased significantly.

While genetic and environmental factors have long been recognized as influential contributors, emerging research has unveiled the profound impact of nutrition on cancer susceptibility, progression, and treatment outcomes. Studies across various cancer types have illuminated the profound effect of the diet on tumorigenesis [3,4]. Melanoma also attracts extensive research efforts in diet and nutritional interventions, as is illustrated by studying the trends in the publication of relevant articles using the Web of Science (WoS) (Figure 1), which covers over 12,000 academic journals and is frequently used by researchers [5]. The search strategy for all relevant articles in the Web of Science Core Collection (WoSCC) was developed after consultations with senior literature search experts. Articles were searched and exported from 1 January 2003 to 1 October 2023, using the following criteria: (TS = (nutrients OR macronutrients OR macronutrient OR diet OR vitamins OR antioxidants OR selenium OR fatty acid) AND melanoma). Among those exported articles, there was an emphasis on population-based cohorts or controlled research among these studies to provide clinical insights with higher translational potential.

The increasing number of articles indicates the necessity for an updated and more comprehensive review of the relationship between nutrients and melanoma. Despite the knowledge about melanoma’s biology, pathogenesis, and development therapies, it is imperative to gain a comprehensive understanding of how dietary compounds influence melanoma. Among those effects, there is a growing interest in the role of diet in redox modulation, which involves the regulation or alteration of the reduction–oxidation balance within melanoma [6]. This aspect, particularly the impact of antioxidant vitamins such as A, C, and E, as well as micronutrients such as selenium, is garnering increased attention. Recent prominent studies have also revealed significant findings in the microbiome of melanoma patients. Consequently, investigating the influence of diet, particularly dietary fiber, on the microbiome represents a promising avenue in melanoma research.

As the skin serves as the body’s largest organ, it is highly susceptible to damage caused by UV in the sun, becoming the main site of primary melanoma. Meanwhile, this unique property makes it highly responsive to dietary influences. The established link between nutrition and skin health [7] underscores the vital role of nutrients in preserving the damage resistance of skin. However, the implications of nutrition extend beyond the skin itself, as nutrient intake has been associated with melanoma susceptibility, progression, and responses to therapy. Further research and clinical trials are warranted to validate these promising findings and unlock the full potential of nutrient integration into melanoma therapy.

This review explores the relationship between nutrients and melanoma, exploring their roles in modulating risk factors and influencing tumor microenvironments, as well as some currently augmented therapeutic strategies. By drawing insights from the intersecting fields of dermatology, oncology, and nutritional science, we aim to provide a comprehensive understanding of the dynamic interplay between nutrient intake and melanoma outcomes. In an era where personalized medicine is gaining momentum, deciphering the intricate mechanisms governing the nutrient–cancer axis holds promise for refining strategies in melanoma prevention and management.

## 2. Nutrients and Melanoma Prevention

### 2.1. Antioxidants

**Figure 2:**
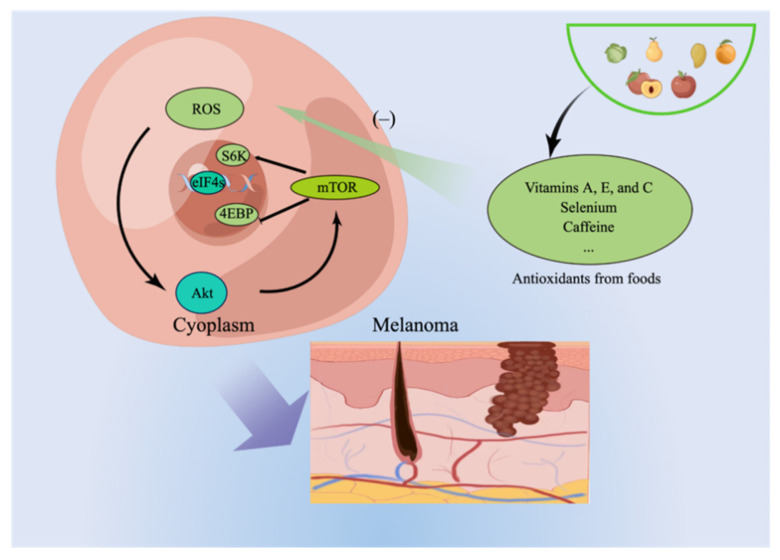
The investigation of ROS scavengers and inhibitors as potential preventive measures for melanoma and protection against skin damage has been prompted by the accumulation of ROS in melanocytes.

Antioxidants hold significant promise as dietary protective factors because they shield melanocytes from damage caused by reactive oxygen species (ROS) [8]. As the origin of melanoma, melanocytes accumulate reactive oxygen species (ROS), driving exploration into ROS scavengers and inhibitors to prevent melanoma and shield against skin damage [9]. Studies have shown that ROS can activate the downstream mTOR signaling pathway and alter glutamine metabolism, causing carcinogenesis [10] (Figure 2). Therefore, foods containing natural antioxidants are potentially protective factors for melanoma development.

#### 2.1.1. Vitamins

Vitamins A, E, and C are antioxidants that neutralize free radicals or even suppress their generation [11]. Several studies have attempted to elucidate the association between various vitamins, the risk of melanoma, and the underlying mechanisms. Studies based on melanoma cell lines or animal models have provided explanations from different perspectives for the mechanisms by which these vitamins reduce the risk of melanoma. This includes vitamin A and its derivatives, which can suppress the proliferation and invasion of melanoma cell lines by regulating the expression of EGFR and ICAM-1 [12]. Vitamins C and E can reduce the damage to the cell genome caused by UV by alleviating oxidative stress [12,13]. Although many in vitro studies have suggested that vitamin supplementation may be associated with a reduced risk of melanoma, epidemiological studies have not supported this evidence due to their controversial results [14,15,16].

In an in vitro study involving cultured keratinocytes, researchers observed that the combined action of vitamins C and E effectively countered the surge in ROS triggered by acute UVB irradiation. This synergistic effect mitigated oxidative stress and protected against UVB-induced apoptosis or cell death [17]. Similarly, in a study using female Swiss albino mice, the carotenoid β-carotene (BC) (a precursor of vitamin A) was found to be effective in preventing skin carcinogenesis induced by benzo[a]pyrene (BP) both in the absence and presence of UV radiation. These carotenoids also showed strong antitumorigenic properties in mammary carcinomas induced by 8-methoxypsoralen (8-MOP) [18]. The study suggested that carotenoids, acting as powerful antioxidants, might prevent carcinogenic risks associated with substances transformed into ultimate carcinogens through oxidative processes.

Moreover, these experimental findings dovetail with the epidemiological evidence, as demonstrated in a population-based case–control study in Northern Italy, highlighting the potential protective effects of dietary vitamin C intake against melanoma risk, particularly among specific demographic groups. In this population-based case–control study involving 380 melanoma patients and 719 matched controls, researchers found that higher dietary vitamin C intake was associated with a reduced risk of melanoma, with odds ratios of 0.86 and 0.59 in the intermediate and highest intake categories, respectively, compared to the lowest class after adjusting for potential confounding factors [14]. This study found that this protective association was particularly notable among young females (<60 years old) and individuals with phototypes II and III.

However, in another population-based study involving two Nurses’ Health Study cohorts of US women, researchers investigated the relationship between vitamin intake (specifically vitamins A, C, and E and individual tocopherols or carotenoids) and the risk of melanoma. They tracked over 162,000 Caucasian women aged 25–77 years for more than 1.6 million person-years. Surprisingly, they found that vitamins A, C, and E and their components were not associated with a decreased risk of melanoma. However, vitamin A intake from foods and supplements showed a protective effect in a subgroup of women who were otherwise at low risk based on non-dietary factors. Notably, higher vitamin C intake from food alone was linked to an increased risk of melanoma and a significant dose–response relationship with the frequency of orange juice consumption [15].

Additionally, a system review that analyzed 13 publications from 9 trials revealed that β-carotene supplementation did not significantly affect the incidence of overall cancers, including pancreatic, colorectal, prostate, breast, melanoma, and non-melanoma skin cancers. These findings cast doubt on the efficacy of β-carotene supplementation for cancer prevention and even suggest potential risks, particularly in specific subgroups [16].

A possible explanation for these contradictory pieces of evidence is that the aforementioned studies used different methods to define vitamin intake, with some quantifying dietary intake while others employed interventions involving supplement intake. Thus, more evidence is needed to draw a conclusion on the association between nutritional supplements and melanoma risk and provide practical clinical suggestions.

#### 2.1.2. Selenium

Selenium is a vital trace mineral. It can be obtained from various dietary sources, such as plants grown in selenium-rich soil, certain meats, fish, and other food items [19]. Selenoproteins, a specific class of proteins, incorporate selenium into their amino acid composition [20]. Recent research has focused on Se’s role in antioxidant selenoproteins, which protect against oxidative stress induced by reactive oxygen and nitrogen species [21]. Conflicting findings exist regarding the relationship between selenium consumption and melanoma risk, as certain human studies indicate a reduced risk with higher selenium intake, while others propose a contrary association [22,23,24].

In a study involving female hairless inbred mice exposed to UV-B irradiation, selenium supplementation with sodium selenite in the drinking water showed a significant dose-dependent protective effect against UV-induced skin cancer. The mice receiving selenium supplementation exhibited a lower incidence of skin tumors than those without supplementation [25]. Additionally, no cases of leukemia were observed in the selenium-supplemented group, whereas some cases occurred in the unirradiated control group.

Another population-based case–control (54 cases, 56 controls) study investigated the connection between selenium exposure and the risk of cutaneous melanoma using multiple exposure indicators. The plasma selenium concentration was strongly associated with an increased risk of melanoma, while toenail and dietary selenium samples showed little association with the disease. The correlation patterns among these exposure indicators varied by disease status [23]. These findings suggest that different selenium exposure indicators can lead to different conclusions regarding melanoma risk, emphasizing the need for further research to understand the complex relationship between selenium exposure and human health.

#### 2.1.3. Caffeine

Biochemical experiments and animal studies have demonstrated that caffeine and various compounds found in coffee can impact several biological processes associated with cancer development. These processes include DNA methylation [26], oxidative damage [27], and apoptosis [28], which play roles in carcinogenesis. For instance, caffeine has been shown to hinder UV-induced cancer development through multiple biological mechanisms. A study combined an in silico data analysis and a cell model to explore the effects of caffeine on melanoma. The research found that caffeine had potent antiproliferative effects on melanoma cells, possibly by promoting melanin production and reducing the secretion of inflammatory signals. Tyrosinase was identified as a key player in mediating caffeine’s effects on melanoma, suggesting its importance in these mechanisms [29].

In a large US cohort study, higher coffee intake was modestly associated with a decreased risk of melanoma. The study involved non-Hispanic white participants and identified 2904 incident cases of malignant melanoma during the follow-up period. The association was particularly significant for caffeinated coffee intake, with a notable risk reduction in those consuming four or more cups daily. However, decaffeinated coffee did not show a significant association with melanoma risk [30]. Further research on coffee intake and its components, especially caffeine, regarding melanoma is recommended.

Another dose–response meta-analysis of prospective cohort studies was conducted to investigate the relationship between coffee consumption (total, caffeinated, and decaffeinated) and melanoma risk. Seven eligible studies involving 1,418,779 participants and 9211 melanoma cases were included. The analysis revealed a significant inverse association between the total coffee consumption and melanoma risk. Specifically, an increase of one cup of coffee per day was linked to a 3% reduction in melanoma risk [31]. While these findings suggest a potential protective effect of coffee intake against melanoma, further research is needed to account for potential confounding factors and explain study variations.

### 2.2. Fatty Acids

Long-chain *n*-3 polyunsaturated fatty acids (LC *n*-3 PUFAs) have emerged as noteworthy dietary components under scrutiny for their potential to prevent malignant melanoma. The initial suggestion of LC *n*-3 PUFAs’ beneficial impact on melanoma risk came from an old epidemiological study [32], which observed lower melanoma rates among the Inuit population, whose fish-rich diet leads to high daily LC *n*-3 PUFA intake.

In an animal study, fat-1 transgenic mice, capable of converting *n*-6 to *n*-3 fatty acids and maintaining a balanced *n*-6/*n*-3 ratio in their tissues independent of diet, were employed to investigate the role of this fatty acid ratio in melanoma formation and growth. The results revealed significant reductions in melanoma development and growth in fat-1 mice, which exhibited higher levels of *n*-3 fatty acids and their metabolite prostaglandin E3 (PGE3) in the tumor and surrounding tissues, along with an upregulation of the *PTEN* gene [33]. In vitro experiments further supported these findings, suggesting that *n*-3 fatty acids, particularly PGE3, exhibit anti-melanoma effects, at least partially through the activation of the *PTEN* pathway.

A case–control study investigated women’s dietary habits with cutaneous malignant melanoma compared to women from the same community in Brisbane, Australia. The study found a significant inverse relationship between a high polyunsaturated fatty acid intake (*p* < 0.01) and melanoma risk (40% reduction in a group of subjects with a higher daily intake of fish (≥15 g vs. <5 g)) [34]. In another prospective population-based study involving 20,785 middle-aged and older women, the relationship between dietary factors, including polychlorinated biphenyls (PCBs) and long-chain *n*-3 polyunsaturated fatty acids (EPA-DHA), and the risk of malignant melanoma was examined. The study identified 67 incident cases of melanoma during 4.5 years of follow-up. Dietary PCB exposure was associated with a four-fold increased risk of melanoma, while EPA-DHA intake was associated with an 80% lower risk [35].

However, some other studies [36,37,38] do not support the hypothesis that PUFAs can reduce the incidence of melanoma. Another Mendelian randomization (MR) study used genetic data from a large melanoma genome-wide association study (GWAS) and SNPs associated with PUFA levels from another GWAS. The results indicated that raising PUFA levels by a significant amount had a negligible effect on melanoma risk, with an odds ratio (OR) of 1.03 and a 95% confidence interval (CI) range of 0.96 to 1.10 [38]. Overall, this MR analysis suggests that PUFAs have either no or minimal effects on melanoma risk. Hence, further research at an advanced level is required to elucidate the association between PUFAs and melanoma.

### 2.3. Flavonoids

Flavonoids represent a vast group of polyphenolic compounds commonly found in vegetables [39]. They are gaining increasing recognition for their anti-tumor properties, making them a subject of interest in cancer prevention and treatment. Investigations into the molecular mechanisms of flavonoids have explored their roles in antioxidant activity, anti-inflammatory responses, immune modulation, anti-proliferation, angiogenesis inhibition, apoptosis induction, and epigenetic modifications [40,41,42].

A study investigated the impacts of various flavonoids on B16 mouse melanoma 4A5 cells. Among these, isoliquiritigenin and butein, significantly inhibited cell growth and induced cell death, characterized by apoptosis. These compounds caused nuclear changes typical of apoptosis, increased the proportion of hypodiploid cells, and influenced the expression of Bcl-2 family proteins. Isoliquiritigenin appeared to induce apoptosis by inhibiting glucose transport and promoting Bax expression, while butein induced apoptosis by downregulating Bcl-2 expression and enhancing Bax expression [43]. These findings suggest distinct mechanisms for chalcone-induced apoptosis in melanoma cells.

Another study used computational methods to elucidate the licorice flavonoids’ (LCFs) molecular mechanism in their anti-melanoma activity. Through network pharmacology, active anti-melanoma components and targets of LCFs were identified. Predictive models were established to assess the compounds’ activity, including 2D-QSAR and 3D-QSAR pharmacophore models [44]. Molecular docking and dynamics simulations were conducted to validate the pharmacophore. While these mechanisms have been studied in in vitro and mouse models, there is currently a shortage of comprehensive epidemiological studies, including melanoma-related ones.

In summary, a wide range of dietary and nutritional compounds have been explored as potential agents for melanoma prevention (Table 1). While many of these compounds exhibit promising in vitro results and have been the subject of extensive epidemiological studies, the lack of high-quality clinical research evidence remains a gap in our understanding. Given the ongoing rise in melanoma rates, the interest in nutritional chemoprevention will persist, and future research may clarify the dietary components’ role in reducing melanoma risk.

## 3. Nutrients and Melanoma Progression

### 3.1. Modulation of Immune Responses by Nutrients

The immune system’s pivotal role in cancer surveillance and control is widely recognized since an optimal nutritional status fosters a robust immune system, bolstering the body’s capacity to defend against infectious agents and eliminate abnormal or depleted cells [52]. Nutrients have consequently emerged as potential regulators of immune responses that influence the progression of melanoma [53].

#### 3.1.1. Vitamin D

Previously, vitamin D was primarily thought to play a role in bone homeostasis [55], although recent research has uncovered its involvement in immunomodulation [56]. Vitamin D has garnered attention for its immunomodulatory properties in multiple cancer types, including regulating T cell function and suppressing inflammatory cytokines [57,58,59]. From a mechanical viewpoint, vitamin D can influence diverse cell types by activating the vitamin D receptor (VDR), similar to how other steroid vitamins such as vitamin A function. Consequently, vitamin D is recognized as a regulator of the immune system, with VDR being present in nearly all immune cells. Vitamin D deficiency has been associated with autoimmune diseases [60], and supplementation has shown positive effects on these disorders. As we delve deeper into the intricate role of vitamin D in melanoma, recent studies have illuminated its potential protective effects against melanoma recurrence and progression, shedding additional light on the immunomodulatory properties of this vital nutrient.

A preliminary retrospective study suggested a potential protective role of vitamin D against melanoma recurrence. In a more extensive prospective cohort study with 872 patients, higher levels of 25-hydroxyvitamin D3 at diagnosis were associated with thinner tumors and improved survival from melanoma, independently of the Breslow thickness. Although self-reported vitamin D supplement use in the retrospective study did not show significant correlations with reduced melanoma relapse risk, the prospective study suggests that vitamin D sufficiency may benefit melanoma patients and those at high risk of melanoma [61]. However, further research is needed to determine its optimal serum levels.

Another study further explored the role of vitamin D signaling through the VDR in melanoma progression. Higher levels of VDR expression were independently protective against melanoma-related death in both primary and metastatic diseases. Tumors with high VDR expression rates showed increased antitumor immune activity and reduced proliferative pathways, particularly Wnt–β-catenin signaling. Vitamin D deficiency was associated with decreased survival in primary melanoma, especially when VDR levels were low [48]. These findings suggest a causal relationship between vitamin D–VDR signaling and melanoma survival, highlighting its role in melanoma progression through immunomodulation.

#### 3.1.2. Zinc

Additionally, zinc, an essential trace element, is integral to the development and function of immune cells and plays a crucial role in both cellular and humoral immune responses regarding its anti-cancer potential [62]. Firstly, zinc deficiency reduces the levels of biologically active thymulin, a hormone vital for T cell function, leading to decreased circulating T cells [63]. Secondly, zinc deficiency disrupts the balance between Th1 and Th2 cell populations [64], inhibiting the production of critical cytokines for tumor suppression such as IFN-γ, IL-2, and TNF-α [65], weakening the immune function. Thirdly, even before affecting thymocyte development, modest zinc deficiencies alter specific thymic mRNA and proteins, impacting cell survival and DNA repair processes [66].

In light of these findings concerning zinc’s pivotal role in the immune function and anti-cancer responses, an intriguing mouse study explored the potential of a zinc chloride fixative paste as an immune adjuvant in melanoma treatment. The experiment involved two types of melanoma in mice, one with low immunogenicity (B16 melanoma) [67] and another with higher immunogenicity (K1735p melanoma) [67]. The tumors were treated with zinc chloride paste and then excised or excised without treatment. Subsequently, the mice were challenged with melanoma cells at a different site to observe tumor growth. The results indicated that zinc chloride fixation significantly reduced the development of K1735p melanoma at the challenge site compared to excision alone [68], suggesting that the zinc chloride fixative paste may enhance the host’s resistance to melanoma by acting as an immune adjuvant, particularly in the case of more immunogenic melanomas.

### 3.2. Nutrients and Angiogenesis

The tumor microenvironment (TME) is a dynamic milieu comprising immune cells, stromal cells, and extracellular matrix components that play a pivotal role in shaping cancer progression [69]. Nutrients can influence various aspects of the TME, ultimately impacting melanoma growth and invasiveness [70]. Angiogenesis, the new blood vessel formation process, is critical for tumor sustenance [71]. Nutrients with anti-angiogenic properties, such as polyphenols found in green tea and certain fruits [72], have garnered attention for their potential to inhibit angiogenesis, thereby hampering melanoma growth.

Polyphenols have demonstrated various health benefits recently, especially their influence on angiogenesis. These compounds can modulate angiogenesis by affecting multiple signaling pathways, including those associated with the activation of receptors by growth factors and protein kinase C tyrosine kinases [73]. They can promote the formation of capillary-like structures by enhancing endothelial cell proliferation, migration, and invasion, or can conversely inhibit angiogenesis steps, leading to the suppression or regression of vascular development [74].

In a study investigating the inhibition of lung metastasis induced by melanoma cells, various polyphenolic compounds, including curcumin, catechin, rutin, epicatechin, naringin, and naringenin, were tested in mice. The oral administration of curcumin and catechin at a concentration of 200 nmol/kg body weight showed significant inhibition of lung metastasis, reducing the number of lung tumor nodules by 80%. Other polyphenols such as rutin, epicatechin, naringin, and naringenin also exhibited inhibitory effects on lung tumor nodule formation, albeit to varying degrees [51]. These findings suggest the potential use of these polyphenolic compounds in preventing the metastatic growth of melanoma cells and increasing the lifespan of treated animals.

Resveratrol, a natural polyphenol with anticancer properties, was also investigated for its potential to inhibit lung cancer metastasis in immunocompetent mice [54]. In vitro experiments showed that resveratrol significantly suppressed the growth of melanoma cell lines. In an in vivo study, resveratrol administered via intraperitoneal injection enhanced the mean survival rate of mice and inhibited lung tumor growth. This effect was associated with increased cytokine CXCL10 and IFN-γ levels, reduced angiogenesis, and decreased tumor infiltration by Tregs. Overall, resveratrol effectively inhibited the lung metastasis of melanoma in this study, making it a promising candidate for further research in melanoma treatment.

While dietary polyphenols hold promise as nutraceuticals with proangiogenic or antiangiogenic properties, further research is needed to determine the optimal concentrations, the specific effects of different polyphenols, and the impacts of their metabolites for their practical and appropriate use in angiogenesis-related applications.

### 3.3. Nutrients and Cell Cycle Progression

Cyclin-dependent kinases (CDKs) tightly regulate cell cycle progression, which must interact with cyclin subunits for activation [75]. G1 phase progression and the G1/S transition are primarily controlled by CDK4 (and CDK6) in conjunction with cyclin D during mid-G1 and later by CDK2 when combined with cyclin E. To become active, CDKs require a complex series of phosphorylation and dephosphorylation events on specific residues [76]. However, in cancer, this regulation is disrupted, leading to uncontrolled cell division and tumor formation. Understanding and targeting these disruptions in the cell cycle is a crucial focus of cancer research and therapy [77].

Various flavonoids were examined for their impacts on cell proliferation and cell cycle progression in melanoma cell lines [78]. Notably, flavonoids such as quercetin and luteolin, which possessed a hydroxyl group at the 3′-position of ring B, led to G1 cell cycle arrest. Conversely, lacking this hydroxyl group, kaempferol and apigenin induced a G2 block. Genistein, featuring a hydroxyl at the 5′-position of ring A, caused G2 arrest, while daidzein, lacking this hydroxyl, led to G1 cell accumulation. The flavonoids inducing G1 arrest inhibited CDK2 activity by approximately 40–60%, while those causing G2/M accumulation did not affect CDK2. Additionally, some flavonoids such as kaempferol, apigenin, and genistein inhibited CDK1 by 50–70%, whereas quercetin, daidzein, and luteolin did not impact CDK1.

While the immune-boosting effects of vitamin D and zinc, the anti-angiogenic properties of polyphenols, and the cell-proliferation-inhibiting effects of flavonoids have received support in various cell and animal studies, there is still a significant lack of population-based research, which limits the clinical translational value. When considering the association between nutrients and melanoma progression, it is essential to emphasize the context-dependent nature of nutrient effects and their potential implications for personalized therapy. However, it is important to note that these interactions demand a cautious evaluation of nutrient–drug interactions and potential conflicts with treatment efficacy. As research into the complex interplay between nutrients and melanoma progression advances, the potential applications extend to therapy development and refinement. The subsequent sections will delve into the practical applications of nutrient-focused adjunctive therapies and highlight the challenges associated with studying these nuanced relationships in clinical settings.

## 4. Nutrients and Adjunctive Melanoma Therapies

The landscape of melanoma therapy has undergone a profound shift with the advent of targeted therapies, immunotherapies, and combination regimens [79]. Within this evolving context, integrating nutritional strategies as adjunctive therapies holds immense promise [80,81]. Nutrients, recognized for their diverse roles in immune modulation, oxidative stress mitigation, and angiogenesis regulation, have attributes that can synergistically enhance the effectiveness of conventional treatments [82]. In addition, nutrients represented by dietary fiber can indirectly affect anti-tumor immunity by affecting the gut microbiome, which has also received extensive attention in melanoma immunotherapy [83]. However, it is vital to clarify that nutrients are not proposed as stand-alone therapies; they serve as an augmentation strategy that complements present treatment modalities.

### 4.1. Vitamin D as a Potential Adjuvant to Surgery

Vitamin D3 exerts pleiotropic effects through its interaction with the VDR, which are significant in the cancer context. These effects encompass cell growth regulation, differentiation, apoptosis, and modulating interactions between tumors and the immune system [84,85]. A few clinical studies have demonstrated a correlation between low plasma vitamin D levels and unfavorable patient outcomes [46,61,86]. Consequently, a study was conducted to investigate whether postmelanoma resection vitamin D supplementation could effectively mitigate the risk of recurrence and enhance the overall prognosis for patients. In this study involving patients with stage II melanoma, the researchers investigated the effects of adjuvant vitamin D3 supplementation versus placebo over three years. They observed a significant increase in serum 25-hydroxy vitamin D (25OHD) levels in the vitamin D3 group compared to the placebo group [47]. Notably, patients with a low Breslow score (<3 mm) showed a more significant increase in 25OHD levels, while those with a Breslow score ≥3 mm had a lower gain. After 12 months, the patients with low 25OHD levels and a Breslow score ≥3 mm had a shorter disease-free survival rate. This suggests a potential role for vitamin D in melanoma prognosis, although more extensive trials are needed to confirm these findings and further explore vitamin D supplementation in melanoma patients.

### 4.2. Dietary Fiber and Melanoma Immunotherapy Response

**Figure 3:**
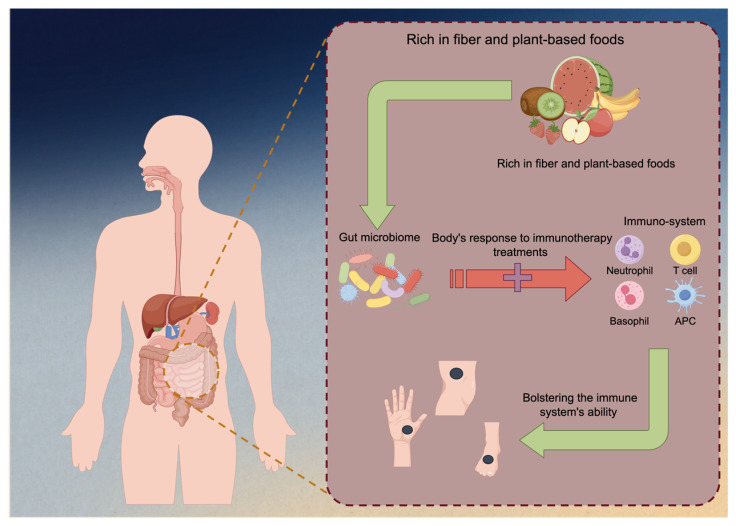
Immunotherapy against melanoma could be enhanced by fiber-rich foods. Diets high in fiber and predominantly plant-based foods have been linked to a more advantageous composition of the gut microbiome, characterized by an increased prevalence of beneficial bacteria. The microbiome profile has the potential to augment the body’s immunotherapy response by fortifying the immune system’s capacity to identify and combat malignant cells.

The emerging research has unveiled a fascinating interplay between diet, gut microbiota, and melanoma immunotherapy. Recent studies suggest that the composition of an individual’s gut microbiome, which is significantly influenced by their dietary habits, can impact the effectiveness of immunotherapy for melanoma and other cancers [87,88,89]. Specific diets rich in fiber and plant-based foods have been associated with a more favorable gut microbiome composition, characterized by a higher abundance of beneficial bacteria [90]. This microbiome profile in turn enhances the body’s response to immunotherapy treatments, potentially by bolstering the immune system’s ability to recognize and attack cancer cells (Figure 3). A study focused on melanoma patients undergoing anti-PD-1 immunotherapy and their gut microbiome discovered significant differences in the diversity and composition of the gut microbiome between responders and non-responders to immunotherapy. Responding patients had higher α-diversity levels and more bacteria from the *Ruminococcaceae* family [91]. A metagenomic analysis also revealed functional distinctions in the gut bacteria of responders, suggesting an enrichment of anabolic pathways. Additionally, immune profiling indicated that responding patients with a favorable gut microbiome displayed enhanced systemic and antitumor immunity.

To further explore whether dietary interventions can affect gut microbes and improve the efficacy of melanoma immunotherapy, another study investigated the impacts of diet and probiotic supplement use on the response to immune checkpoint blockade (ICB) treatment in melanoma patients. Higher dietary fiber intake was associated with improved progression-free survival in patients receiving ICB (*n* = 158, median PFS 17 vs. 23 months) [89]. Interestingly, the most significant benefit was observed in patients with adequate dietary fiber intake who did not use probiotics. These findings were consistent with preclinical models [92], suggesting that a low-fiber diet or probiotic use might impair the response to ICB therapy.

These intriguing findings highlight the importance of considering the diet and gut microbial health as potential adjuncts to melanoma immunotherapy, opening exciting avenues for personalized cancer treatment approaches that harness the power of both medicine and nutrition.

### 4.3. Targeted Nutrient Deprivation (TND) Therapy in Melanoma

Targeted nutrient deprivation (TND) is an emerging approach in cancer therapy [93,94,95], including melanoma. TND exploits the distinct amino acid requirements of normal and cancer cells. In humans, amino acids are categorized as either essential or non-essential. Essential amino acids (EAAs) must be obtained through the diet, while non-essential amino acids (NEAAs) are typically synthesized within the body through their own biochemical processes [96]. However, cancer cells often exhibit altered metabolic patterns and rapid proliferation, making it challenging to produce an adequate supply of NEAAs internally [97]. Consequently, NEAAs become vital nutrients for tumor cells, reliant on external sources for their provision. This forms the foundation of the TND approach.

By targeting the metabolic dependencies of melanoma cells, TND aims to weaken their resilience and enhance the effectiveness of present therapies, such as immunotherapy or targeted therapies. A study investigated the effectiveness of combining recombinant methioninase (rMETase), which targets methionine dependence in cancer cells, with a first-line melanoma drug temozolomide (TEM) in a patient-derived orthotopic xenograft (PDOX) mouse model of BRAF V600E mutant melanoma. Four groups of melanoma PDOX mice were studied: an untreated control, TEM alone, rMETase alone, and the combination of TEM and rMETase. All treatments led to a reduction in tumor growth compared to the untreated control [98]. Notably, the combination therapy of TEM and rMETase demonstrated superior efficacy compared to either treatment alone. This suggests the potential clinical development of rMETase combination therapy, particularly in challenging cancers such as melanoma, where it may enhance the effectiveness of first-line treatments.

Another case study experimentally combined TND and melanoma immunotherapy. A 65-year-old patient with advanced melanoma, who had not responded to two different immunotherapy strategies, was enrolled in a phase I study and treated with pegylated recombinant arginase (BCT-100). Remarkably, the patient achieved sustained complete remission lasting over 30 months with no severe side effects [99]. An analysis of the tumor revealed a state of arginine auxotrophy, where the cancer cells could not produce arginine. Targeting arginine metabolism with therapeutic arginase proved to be an effective salvage therapy for this patient, suggesting a promising avenue for treating melanoma cases that do not respond to conventional immunotherapy. While this field of research is still evolving, TND holds promise as a complementary strategy to combat melanoma and improve treatment outcomes.

Several approaches to using nutrients for the adjuvant treatment of melanoma have received clinical research support, including the use of vitamin D to reduce postoperative recurrence in melanoma patients and the use of a high-fiber diet to enhance the effectiveness of immunotherapy. Additionally, the research related to TND therapy is progressing rapidly. It is worth mentioning that the success of nutrient-focused adjunctive therapies rests upon meticulous patient assessments and the optimization of the nutrient status [100]. The individual nutrient requirements vary based on age, overall health, and treatment regimens. Rigorous monitoring of nutrient levels, tailored dietary recommendations, and the judicious use of nutritional supplements contribute to improved treatment responses [101]. Furthermore, patient education on dietary modifications and nutritional supplementation empowers individuals to actively participate in their treatment journey, potentially leading to more favorable therapeutic outcomes. In exploring nutrient integration into melanoma therapy, it is paramount to emphasize interdisciplinary collaboration. Dermatologists, oncologists, nutritionists, and researchers must forge synergies to navigate the complexities of nutrient–drug interactions and harness the potential benefits of adjunctive therapies [102]. The fusion of medical expertise and nutritional insights offers an integrative platform that promises to optimize melanoma treatment strategies and contribute to the advancement of oncology care.

## 5. Conclusions

This narrative review has outlined the relationship between nutrients and melanoma, spanning from prevention to their role as therapeutic adjuvants. The article begins by examining the functions and underlying mechanisms of various nutrients, including antioxidants (namely vitamins A, D, C, and E; selenium; caffeine), polyunsaturated fatty acids, and flavonoids, in reducing melanoma risk. Among these nutrients, caffeine holds the most promising potential for melanoma protection, as it is supported by multiple cohort studies. While there is robust evidence from cellular and animal studies regarding the immunomodulatory properties of vitamin D and zinc, the anti-angiogenic potential of polyphenols, and the cell growth-inhibiting effects of flavonoids, their practical significance in clinical settings is greatly limited due to the scarcity of human-based research. As for the utilization of nutrients in adjuvant melanoma treatment, various approaches have gained clinical research support, including using vitamin D to reduce postoperative recurrence among melanoma patients and adopting a high-fiber diet to enhance the effectiveness of immunotherapy. We believe that there is substantial in vivo and in vitro evidence supporting reductions in melanoma risk from moderate caffeine intake, as well as the improvement of treatment outcomes and prognosis through vitamin D supplementation and dietary fiber use. Therefore, these can potentially be recommended for melanoma patients.

Currently, there is a degree of inconsistency in the research findings regarding the role of nutritional components in melanoma prevention and treatment. This disparity may be attributed to variations in how intake is defined, patient population heterogeneity, and the absence of large-scale retrospective studies. Considering the potential demonstrated by these nutrients in in vitro studies and certain population-based research, it is worthwhile to allocate more resources to well-designed large-scale clinical trials and retrospective studies. This approach aims to minimize the disease burden associated with melanoma to the greatest extent possible. Additionally, the advancement of melanoma therapy necessitates interdisciplinary collaboration. The fusion of medical expertise with nutritional insights offers an integrative platform that optimizes melanoma treatment strategies.

Our study innovatively elaborates on the impacts of nutrients on melanoma prognosis and treatment, updating the latest research in the field, which may serve as an inspiration for further studies. However, this review also has certain limitations, including the inability to conduct a comprehensive quantitative analysis of the results due to research heterogeneity issues. Additionally, we did not delve into the effects of specific dietary patterns (such as the Mediterranean diet or DASH diet) on melanoma, as their definitions are not uniformly established.

## Figures and Tables

**Table 1:** Various nutrients and their effects and mechanisms on melanoma.

Nutrients		Effects	Mechanism	References
Vitamin	A	Potentially reduces risk of melanoma	(1) Regulation of EGFR expression (2) Intercellular adhesion molecule gene I (ICAM-1) is transcriptionally regulated by retinoic acid in melanoma cells	[13,15,45]
	D	Slows melanoma progression through immunomodulation	Interacts with VDR and reduces proliferative pathways, particularly Wnt–β-catenin signaling	[46,47,48]
	C and E	Potentially reduces risk of melanoma	This collaborative effect effectively alleviated oxidative stress and provided protection against UVB-induced apoptosis or cellular demise	[15,17,49,50]
Selenium		Controversial impact on melanoma effects	Works against oxidative stress induced by reactive oxygen and nitrogen species	[14,22,23,24]
Zinc		Slows melanoma progression by enhancing immune response	Assists the production of critical cytokines for tumor suppression and maintain the balance between Th1 and Th2 cell populations	[51,52,53]
Caffeine		Potential protective effect against melanoma	Impacts several biological processes associated with cancer development, including DNA methylation, oxidative damage, and apoptosis	[28,29,30]
Fatty acid		No or minimal effects on melanoma risk	Activation of the PTEN pathway	[33,35,38]
Flavonoids		Induction of apoptosis in melanoma cells	Antioxidant activity, anti-inflammatory responses, immune modulation, anti-proliferation, angiogenesis inhibition, apoptosis induction, and epigenetic modifications	[40,41,42]
Polyphenols		Anti-angiogenic effect during melanoma progression	Inhibition of the activation of receptors of the protein kinase C tyrosine kinase pathway	[9,51,54]

## Data Availability

Not applicable.

## Abbreviations

β-carotene, BC; Cyclin-dependent kinases, CDKs; Essential amino acids, EAAs; Genome-wide association study, GWAS; Immune checkpoint blockade, ICB; Licorice flavonoids, LCFs; Mendelian randomization, MR; Prostaglandin E3, PGE3; Polychlorinated biphenyls, PCBs; Reactive oxygen species, ROS; Targeted Nutrient Deprivation, TND; Tumor microenvironment, TME; Vitamin D receptor, VDR.
